# Transport and Application Layer DDoS Attacks Detection to IoT Devices by Using Machine Learning and Deep Learning Models

**DOI:** 10.3390/s22093367

**Published:** 2022-04-28

**Authors:** Josue Genaro Almaraz-Rivera, Jesus Arturo Perez-Diaz, Jose Antonio Cantoral-Ceballos

**Affiliations:** Tecnologico de Monterrey, School of Engineering and Sciences, Monterrey 64849, Nuevo Leon, Mexico; a00821189@tec.mx

**Keywords:** class balancing, DDoS attacks, deep learning, DoS attacks, intrusion detection system, IoT networks, machine learning

## Abstract

From smart homes to industrial environments, the IoT is an ally to easing daily activities, where some of them are critical. More and more devices are connected to and through the Internet, which, given the large amount of different manufacturers, may lead to a lack of security standards. Denial of service attacks (DDoS, DoS) represent the most common and critical attack against and from these networks, and in the third quarter of 2021, there was an increase of 31% (compared to the same period of 2020) in the total number of advanced DDoS targeted attacks. This work uses the Bot-IoT dataset, addressing its class imbalance problem, to build a novel Intrusion Detection System based on Machine Learning and Deep Learning models. In order to evaluate how the records timestamps affect the predictions, we used three different feature sets for binary and multiclass classifications; this helped us avoid feature dependencies, as produced by the Argus flow data generator, whilst achieving an average accuracy >99%. Then, we conducted comprehensive experimentation, including time performance evaluation, matching and exceeding the results of the current state-of-the-art for identifying denial of service attacks, where the Decision Tree and Multi-layer Perceptron models were the best performing methods to identify DDoS and DoS attacks over IoT networks.

## 1. Introduction

Distributed Denial of Service (DDoS) attacks are one of the main threats to network systems, affecting the applications and devices that rely on them. DDoS attacks consist of grouping multiple devices against one target, preventing legitimate users to access services such as email and websites. They represent the most common and critical attack against and from Internet of Things (IoT) devices, Cloud Computing, and fifth-generation (5G) communication networks [1].

DDoS attacks can be categorized into two main classes according to the size of the traffic they generate: high-rate and low-rate attacks. The behavior of low-rate attacks is extremely inconspicuous since they behave similarly to legitimate traffic and can account for about 10–20% of the total normal network traffic [2]. Although the average traffic of low-rate attacks is small, they can potentially not only reduce the quality of service of the target but also stop the service completely [2]. This is achieved by an attacker sending periodically pulsing data, instead of continuous flows [3]. Examples of low-rate denial of service attacks are GoldenEye, Slowloris, and R.U.D.Y. (R-U-Dead-Yet?) [4].

In contrast to low-rate attacks, high-rate DDoS attacks employ an approach of high-rate packet transmission, where the statistical changes in the behavior can be used to distinguish them from the normal data flows [3]. High-rate attacks violently exhaust the resources and the capacity of the network, making the victim unresponsive in a short period of time [2]. Examples of high-rate denial of service attacks are SYN Flood and UDP Flood [2].

According to the Kaspersky Q2 2020 DDoS Attacks Report [5], in the second quarter of 2020, the number of DDoS attacks slightly increased compared to the first quarter of the same year (from 302.08% to 316.67%) and more than three-fold compared with the data for the same period in 2019. In addition, the Kaspersky Q3 2021 DDoS Attacks Report [6], when compared to the same quarter of 2020, shows that the total number of DDoS attacks increased by nearly 24%, and the total number of advanced and targeted DDoS attacks increased by 31%.

IoT devices are susceptible to hijacking for conducting high-scale attacks without the device owner’s knowledge, which may lead to the creation of botnets [7]. These vulnerabilities can be due to weak passwords and to the lack of robust security standards from manufacturers [7]. For instance, the Mirai botnet unleashed massive DDoS attacks on major websites from millions of compromised devices in 2016, showing the power of IoT attacks [8]. Another example is Mozi, which emerged in 2019, and is the most active Mirai-type variant, controlling approximately 438,000 hosts, which target routers and cameras, and in 2020 accounted for 89% of the total IoT attacks detected by IBM for the year [8]. These data corroborate the need for computer systems and security mechanisms capable of protecting the IoT infrastructure, which is even more evident because everyday more and more devices are connected to and through the Internet (by 2025, there will likely be more than 27 billion IoT connections [9]). From smart homes to industrial environments, IoT is an ally in our daily activities, where some applications are critical. To achieve this protection, some approaches must be taken, such as Intrusion Detection Systems (IDS) [10] and Intrusion Prevention Systems (IPS).

This research presents a novel smart IDS, based on Machine Learning (ML) and Deep Learning (DL) models using the Bot-IoT dataset [11], published in 2019. This dataset was created with the simulation of IoT sensors emulating a smart home arrangement: a weather station, a smart fridge, motion activated lights, a remotely activated garage door, and a smart thermostat. This dataset was chosen because it is a state-of-the-art dataset observed in other approaches for protecting IoT devices [12,13,14,15,16,17] since it contains realistic normal and attack traffic. Furthermore, this dataset has a subcategory field which enables multiclass classification, and it presents information of DDoS and Denial of Service (DoS) attacks generated using GoldenEye [18] for Application layer protocols (HTTP) and Hping3 [19] for Transport layer protocols (UDP, TCP). The Argus tool [20] was used by the dataset authors, after collecting the pcap files, to generate the network flows and produce the features.

SYN flooding is one of the attack variants contained in the Bot-IoT dataset, and according to the Kaspersky Q3 2021 DDoS attacks report [21], it is the method used in 51.63% of the attacks. Flooding DDoS attacks based on UDP finished second with 38%, and those based on TCP remained third with 8.33%. DDoS attacks based on HTTP finished in fourth place with 1.02%. This distribution of denial of service attacks by type motivates us to create models suitable for testing in Transport and Application layers, since they represent at least 47% of the whole attack distribution, and when accompanied with the SYN flooding variant from the Bot-IoT dataset, we cover nearly 99%.

The total amount of records in the Bot-IoT dataset exceeds 72 million [22], whilst 5% of these data (i.e., over 3 million records) are used in [11] by the authors of the dataset. However, the Bot-IoT dataset suffers from severe class imbalance [23], with just a few thousands (about 9000) normal flows, a limitation that we address in our research. We can summarize the main contributions of this work as follows:

- Anomaly detection models that match and exceed the performance of the current state-of-the-art for identifying specific denial of service attacks categories, using three different feature sets;
- A comprehensive evaluation of the classification and time performance of several Machine Learning and Deep Learning models with three different feature sets, which led to the discovery that it is not necessary to use the Argus flow data generator for any future online implementation based on the Bot-IoT dataset;
- A suitable way to address the Bot-IoT dataset bias problem without adding class weights and without generating synthetic data.

This paper is structured as follows: the literature review is presented in Section 2. Section 3 describes the methodology for the creation of the novel Artificial-Intelligence-based Intrusion Detection System here proposed. The analysis and evaluation of the results are shown in Section 4. Finally, in Section 5, we present the conclusions and future work.

## 2. Related Work

In the original publication of the Bot-IoT [11], its authors evaluated their work by training three different models: one Machine Learning model based on a Support Vector Machine (SVM) with a linear kernel and two Deep Learning models using a simple Recurrent Neural Network (RNN) and a Long-Short Term Memory (LSTM) architecture.

The performance of the models was evaluated with two different sets of features: The first of them used the 10 best features (selected from a filter with Correlation Coefficient and Joint Entropy), and the second one used all 35 features.

For multiclass classification of all the attacks in the Bot-IoT dataset, the best accuracy was of 99.988% with an SVM using all the 35 features. In terms of exclusively DDoS and DoS attacks, the work only reports binary classifications (e.g., Normal flows vs. DDoS HTTP), obtaining the maximum accuracy of 99.999% for Normal flows vs. DDoS UDP with an RNN.

Nevertheless, the dataset was unbalanced [23], which may have positively affected the identification of attacks (i.e., the majority class) due to data bias. This is one of the opportunities we address in this research.

The work in [12] also used 5% of the Bot-IoT dataset and presented 7 different Deep Learning models, including RNNs, achieving a maximum sensitivity of 96.868% for Normal flows vs. DoS HTTP. With respect to DDoS, the maximum sensitivity was for Normal flows vs. DDoS UDP, with 96.666% accuracy.

In [13], the authors applied different Random Forest configurations, tuning the depth and the number of trees. The authors proposed 6 different feature sets (from 4 to 8 features, such as IP, port, and timestamp), and compared their accuracies with the 10 best features set and the SVM in the Bot-IoT paper [11]. The accuracy of the SVM with the 10 best features set is 88.372%, while the accuracy of the proposed Random Forest (with the 6 different feature sets) is 100%. Nevertheless, it is important to note that the experiments in [13] not only considered either DDoS or DoS attacks but also included other types of attacks, such as data ex-filtration and service scanning. No other models were presented by the authors, only Random Forest with a small number of features, which might lead to a loss of information. With respect to time performance, the authors only evaluated the effects of the Random Forest sizes on run-time overheads to classify a single data packet.

In [14], a packet-level model based on Deep Learning was proposed using Feed Forward Neural Networks (FFNN) for binary and multiclass classification with the Bot-IoT dataset. The four categories of attacks were DoS, DDoS, reconnaissance, and information theft in order to differentiate them from the normal traffic. Confusion matrices were generated, and the accuracy, precision, recall, and F1 score metrics were used for performance evaluation. With respect to only DoS and DDoS attacks, the proposed model presented all accuracies above 99% in binary classification (e.g., Normal flows vs. DDoS TCP) and an accuracy of 99.414% for multiclass classification. In order to deal with the unbalanced nature of the dataset, class weights were introduced to the training data, so the class with a smaller number of samples received a higher weight value. However, this technique could introduce the risk of over tuning, resulting in weights that may not generalize optimally [14].

In [15], the Bot-IoT dataset was used to validate a new feature-selection algorithm based on the Area Under the Curve (AUC) metric. A feature set of five variables was selected as the best one, and the mean and the standard deviation of the duration of the aggregated records were two of those features. Only four Machine Learning models were applied: Decision Tree, Naive Bayes, Random Forest, and SVM. The accuracy, precision, recall, and specificity metrics were used for performance evaluation. In terms of results, Random Forest and Decision Tree showed an accuracy of 100% for HTTP, TCP, and UDP denial of service attacks detection. This paper presented a solution for the problem of selecting effective features for accurate attack detection in IoT networks. The AUC metric is useful for dealing with imbalanced datasets [24]; nevertheless, the research work neither evaluates Deep Learning models nor presents a performance evaluation metric, such as the average of flows per second each model can process, which is relevant to evaluate the feasibility of the real-time implementation of their proposed best models.

The work in [16] presented a novel use of Gated Recurrent Units (GRU) in the Bot-IoT dataset. GRUs aim to solve the vanishing gradient problem in a standard RNN [16] by using update and reset gates. The proposed model used only 125,971 samples from the original Bot-IoT dataset in order to conduct a fair comparison and to have the same size as the NSL-KDD dataset [25], obtaining an accuracy of 99.76% for Normal vs. Attack traffic identification, with no exclusivity for either DDoS or DoS attacks.

In [17], the Bot-IoT dataset was used for conducting binary and multiclass classification tasks, with balanced and unbalanced representations of it, where the class balancing technique used was based on weights, as seen in [14]. As mentioned by the authors in [17], they used the default values of the hyper parameters for each classifier, as provided by Scikit-Learn [26] and Keras [27]. In terms of performance metrics, they present indicators such as accuracy and F1 score; however, the authors did not present an evaluation of the models feasibility in a real-time scenario (e.g., by evaluating time performance). From the original Bot-IoT dataset that has 35 variables, the authors removed columns with missing values, as well as columns that contain text and columns they considered to be irrelevant. Their complete dataset had 19 variables, where features such as the timestamps and the Argus sequence number remained. For training and testing, they applied a data split of 80% and 20%, respectively, with no percentage reported for a validation set. For the weighted datasets, the Artificial Neural Network (ANN) was the most outstanding model, with a stable accuracy of 99% for binary classification in DDoS and DoS attacks protocols. For the multiclass classification, the authors presented an overall accuracy with all the attacks types contained in the Bot-IoT dataset, where the ANN kept in first place had an accuracy of 97%. The authors stated they did not train Deep Learning models.

In [23], the authors recognized the need for class balancing in the Bot-IoT dataset. This study showed that the majority classes belong to the attack types, while the normal traffic is part of the minority classes with only 9515 samples (accompanied with information theft, which has 1587 samples), resulting in a ratio of normal to malicious traffic of 1:7687 [23]. An imbalanced dataset may lead to problems such as poor accuracy and/or bias towards the majority class in the results obtained. Specifically, talking about DDoS and DoS attacks, the normal to attack traffic ratio for DoS is 1:459 (i.e., 9515 to 33,005,194 flows), and the ratio for normal to DDoS is 1:4038 (i.e., 9515 to 38,532,480 flows) [23]. Thus, the Bot-IoT dataset seems to be better suited to distinguish between a DoS and a DDoS attack [23], since these categories have similar number of samples (i.e., about 38 million for DDoS and 33 million for DoS).

In order to deal with imbalanced datasets, resampling techniques can be applied to ameliorate this problem. When oversampling, minority class instances are created, either by duplicating elements or by creating new ones synthetically from a similar distribution. The latter technique can be achieved using the Synthetic Minority Oversampling Technique (SMOTE), where, depending on the amount of oversampling required, neighbors from the *k* nearest neighbors are randomly chosen, with one sample generated in each one’s direction [28]. When undersampling, samples from the majority class are removed, which can cause loss of information. We propose to tackle the data bias problem of the Bot-IoT dataset by selecting random consecutive flows per each DDoS/DoS attack type to preserve the temporal behavior of the attacks whilst not altering the network traffic collected from the realistic testbed configuration used to design the Bot-IoT dataset.

In addition, we carry out a comprehensive experimentation specialized in normal flows vs. DDoS and DoS attacks in binary and multiclass classifications with three feature sets to evaluate how different flow processors could be used in a real-world scenario. Dividing our experiments into binary and multiclass classifications allows us to evaluate the detection and identification of network traffic, respectively, leveraging the categories and subcategories present in the Bot-IoT dataset. Likewise, we compare seven Machine Learning and Deep Learning models, using popular metrics such as accuracy and precision. Furthermore, we include time performance to analyze the feasibility of implementation of our smart IDS in a real-time environment.

Table 1 presents a summary comparing the related work with our approach. We present this comparison across six relevant criteria to describe the position of our work and how it stands out from the current state of the art. As can be seen, our work is one of the two that evaluates time performance and is the only one that also tackles the class balancing problem of the Bot-IoT dataset whilst using different feature sets, evaluating ML and DL models, all at a flow-level detection.

## 3. Methodology

In this section, we describe the methodology we followed to create our balanced dataset, as well as the feature standardization process we applied to help convergence in the different classifiers. Likewise, we present a summary of the ML and DL models parameters to conduct our experiments with each of the three feature sets and define the different performance metrics to evaluate our results.

**Figure 1:**
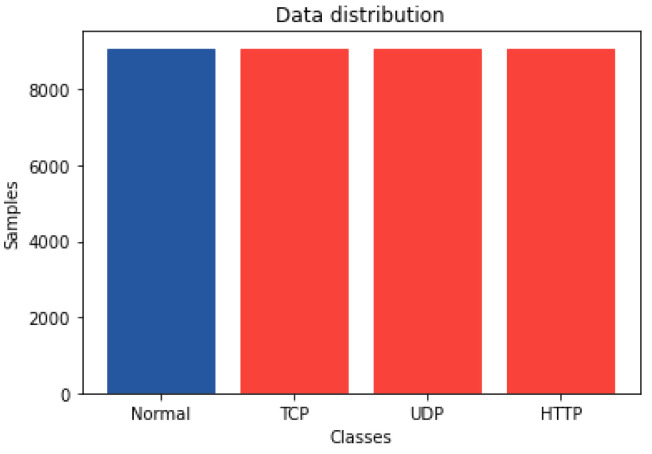
Data distribution plot for multiclass classification.

**Figure 2:**
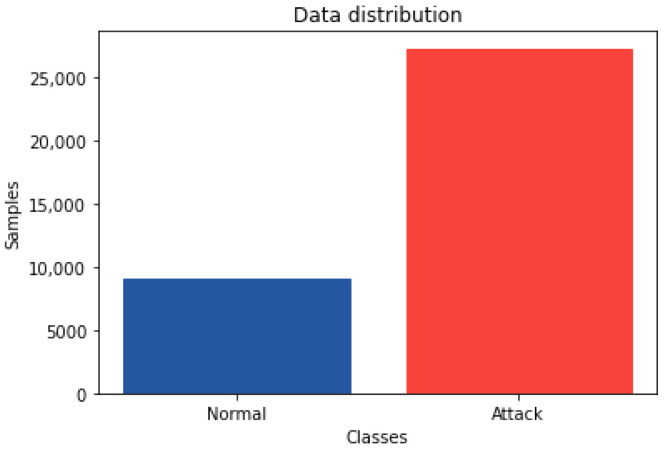
Data distribution plot for binary classification.

The labeled CSV files were downloaded from [22]. A total of 9085 samples were extracted for the normal class. We selected items in the majority class (the attacks) by randomly choosing sections of consecutive flows for each DDoS/DoS attack type in the same proportion to the normal samples to keep a balanced ratio. Figure 1 shows that we achieved the same number of flows for each of the classes for the multiclass classification, where UDP, TCP, and HTTP, are samples from both DDoS and DoS attacks. See Figure 2 for the distribution for binary classification. In the end, the complete dataset size was of 36,340 samples.

In order to design our models, we selected three different feature sets from the original Bot-IoT dataset that has 35 variables. We followed this approach in order to evaluate how the records timestamps affect in the models predictions and to avoid dependencies produced by the Argus flow data generator [20] (so that more flows processors could be used either in a simulated or in a real network implementation, such as CICFlowMeter [29] or Flowtbag [30]).

As seen in Table 2, all the feature sets share the same statistical variables (i.e., rates, mean, maximum, minimum, etc.). The first feature set we tried was selected to evaluate the impact of the timestamps and the Argus sequence number on the classification results. The second feature set removed the timestamps because we argue that the model could memorize these features, which may lead to poor generalization in a real-time scenario. Likewise, we removed the Argus sequence number to avoid dependencies with this parser. Finally, in the third feature set, we kept the Argus sequence number in agreement with the current state-of-the-art (that use this feature) to evaluate how it affects the classifications excluding only the timestamps.

The three feature sets ranged between 15 and 18 variables, which were selected after dropping columns with missing values and choosing statistical features to capture the traffic behavior. No more feature removal was applied in order to capture the greatest amount of information possible. See Table 3 for the description of the variables. It is relevant to note that 8 of the variables in the 10 best feature set identified in [11] were included in the first and the third feature sets we proposed, and 7 of those 10-best variables were in the second feature set.

**Figure 3:**
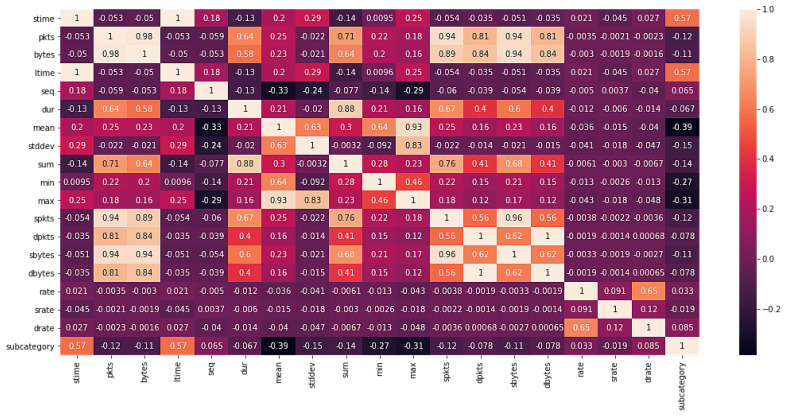
Correlation matrix for multiclass classification.

**Figure 4:**
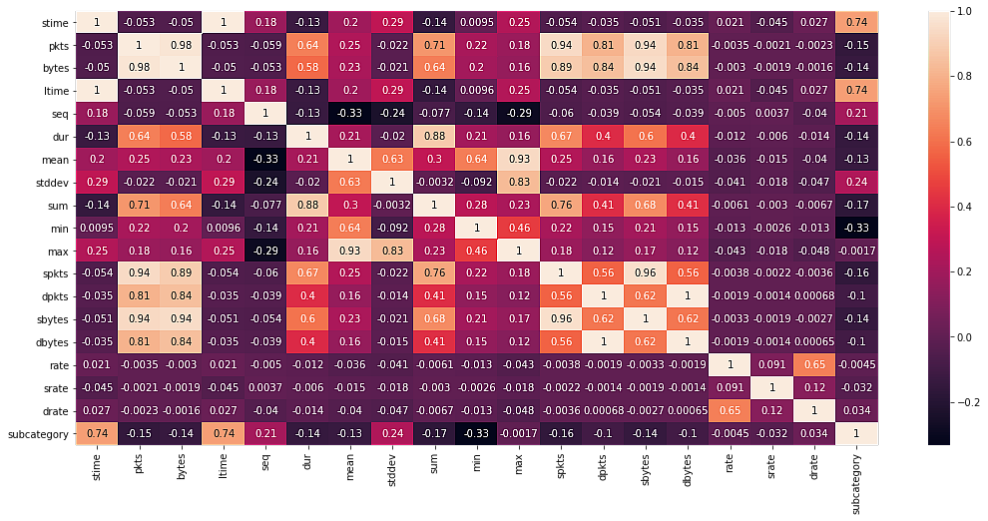
Correlation matrix for binary classification.

The correlation matrix for the multiclass classification task is shown in Figure 3 and the binary classification in Figure 4. Here, the subcategory represents the class to predict. Since we wanted to see the linear relation between our variables (where all are numerical), we calculated these matrices using the Pearson’s correlation coefficient, resulting in values between −1 and 1, where positive values indicated a pair of features that increase or decrease together, and negative values indicated that the increase in one variable implies the decrease in another variable (and vice versa).

To help convergence, the features were standardized by subtracting the mean (centering) and dividing by the standard deviation (scaling), resulting in a set of values whose mean was 0, and the standard deviation was equal to 1. See Equation (1) for the formula:

(1) $$x^{\prime }=\frac{x-mean}{stddev}$$

The dataset split for all the Machine Learning and Deep Learning models was 80% for training, 10% for validation (tuning hyper parameters), and 10% for testing. Given our total number of samples, we decided to create separate sets for training, validation, and testing instead of using other alternatives such as k-fold cross-validation.

All the ML models (i.e., Support Vector Machines, Decision Trees, and Random Forests) were built using Scikit-Learn [26] and the DL models (i.e., RNN, GRU, LSTM, and Multi-layer Perceptron [MLP]) using PyTorch [31]. Confusion matrices were generated and accuracy, precision, recall, and F1 score metrics, in addition to time performance as proposed in [32], were used for evaluation and models benchmark. See Equations (2)–(5), for these metrics’ definitions:

(2) $$Accuracy=\frac{TP\;+\;TN}{TP\;+\;TN\;+\;FP\;+\;FN}$$

(3) $$Precision=\frac{TP}{TP\;+\;FP}$$

(4) $$Recall=\frac{TP}{TP\;+\;FN}$$

(5) $$F1\;score=2*\frac{Precision\;*\;Recall}{Precision\;+\;Recall}$$

Table 4, Table 5 and Table 6 show the parameters for the ML models using the three feature sets. With respect to the DL models, all of them share the characteristics presented in Table 7, where the input size varies according to the feature set (i.e., 18 for the first set, 15 for the second one, and 16 for the third one). The hyper parameters for both Machine Learning and Deep Learning were chosen after a process of systematic tuning. In this regard, the best max depth obtained for the Decision Tree, in both binary and multiclass classification, was correspondingly used as max depth for the Random Forest sub-estimators. Likewise, we report the number of trees that led us to optimal balance between accuracy and run-time. It should be noted that the Decision Tree implementation Scikit-Learn uses is an optimized version of the CART (Classification and Regression Trees) algorithm [33].

Next, the results and discussion for the experiments are presented.

## 4. Experimental Results and Discussion

The results for multiclass and binary classification (for the first feature set) are presented in Table 8 and Table 9, respectively; for the second feature set, we display the results in Table 10 and Table 11; finally, Table 12 and Table 13 show the results for the third feature set. From these results, it can be seen that Decision Tree and Random Forest have the best performance for both classification tasks in the three distinct feature sets, outperforming the DL models. On the other hand, SVM is the poorest-performing model (in agreement with previous work in [11] with the 10-best feature set).

Our results show that Machine Learning models such as Random Forest and Decision Trees show strong performances that are marginally better than that presented from the Deep Learning models. Since all the results show a similar order of magnitude, we argue that given the relative small amount of features in all our datasets, Decision Tree methods show a robust performance that does not learn to depend on one particular feature, thus generalizing better. This also shows that traditional ML models are reliable and should not be discarded without proper evaluation, such as the one we carried out here, and particularly when using tabular data.

Our sampling methodology allowed us to use standard cost functions without weighting techniques, whilst addressing the balancing problem in the Bot-IoT dataset in contrast to what is commonly performed in the current state-of-the-art (e.g., in [14]), which may lead to over tuning. Likewise, our feature sets included more characteristics compared to [13], capturing more information whilst reducing the amount of manual feature engineering, in agreement with the ethos of current Machine Learning practices. With this, we presented comprehensive tests with both Machine Learning and Deep Learning models (compared, for instance, to [17], where only Machine Learning models were presented by the authors).

Software Defined Networks (SDNs) [34] represent one of the best options to implement smart Intrusion Detection Systems due to their relevance for data centers, 5G technology, and the ease of integration of IoT devices to these type of networks. SDNs are capable of achieving higher system flexibility and scalability, separating the data plane and the control plane to provide a dynamic network structure [35]. All the network control functions, such as traffic monitoring, take place in a software-based controller [1], which can either be physically centralized or distributed but logically centralized [36]. This flexibility on global network monitoring and network configuration enables the implementation of detection and mitigation mechanisms against cyberattacks [1].

Given the importance of real-time hardware implementations, we consider it to be relevant to evaluate the time performance of each model for classifying network traffic. As proposed in [32], we calculate the average number of flows per second our anomaly detection methods can classify. This experimentation was conducted on a MacBook Pro with Apple M1 Chip and 16 GB RAM for both the multiclass and the binary classification models, with the three feature sets. See Table 14 and Table 15 for the first feature set; Table 16 and Table 17 for the second feature set; and Table 18 and Table 19 for the third feature set.

From the real-world scenario tested in [32], on regular days, around 500 flows/s passed through the network collector, while in dense traffic situations, it achieved peaks of a maximum of 1681 flows/s. Then, for the first feature set, from Table 14 and Table 15, all the models, except for Random Forest in binary classification, are capable of analyzing the amount of flows/s required on heavy traffic days; whilst for the second feature set (Table 16 and Table 17), all the models, except for SVM in both classification tasks, achieve the maximum peak. Finally, the results for the third feature set (Table 18 and Table 19) show that all the models, except for SVM and Random Forest in multiclass and binary classification, achieve the maximum amount of flows/s discussed.

**Figure 5:**
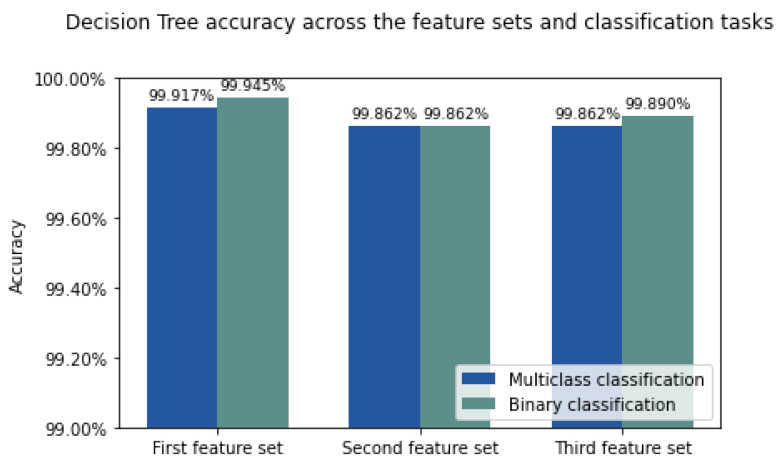
Decision Tree accuracy, as the best model, across the three different feature sets for binary and multiclass classifications.

**Figure 6:**
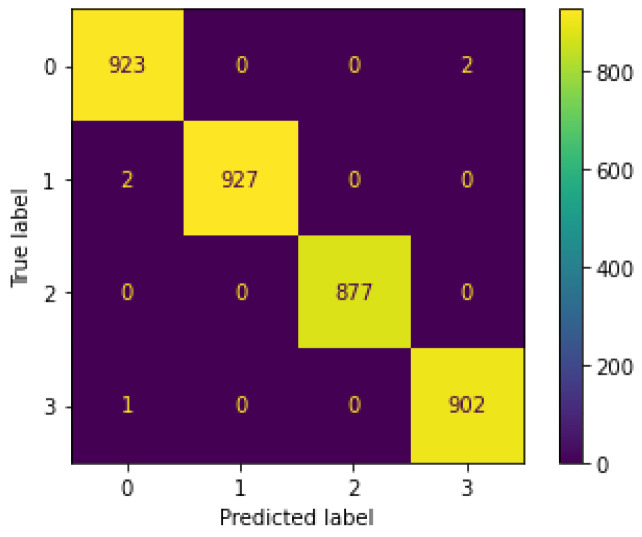
Confusion matrix for Decision Tree multiclass classification, using the best feature set. The numbers in the axes mean 0 for Normal class, 1 for UDP class, 2 for TCP class, and 3 for HTTP class.

**Figure 7:**
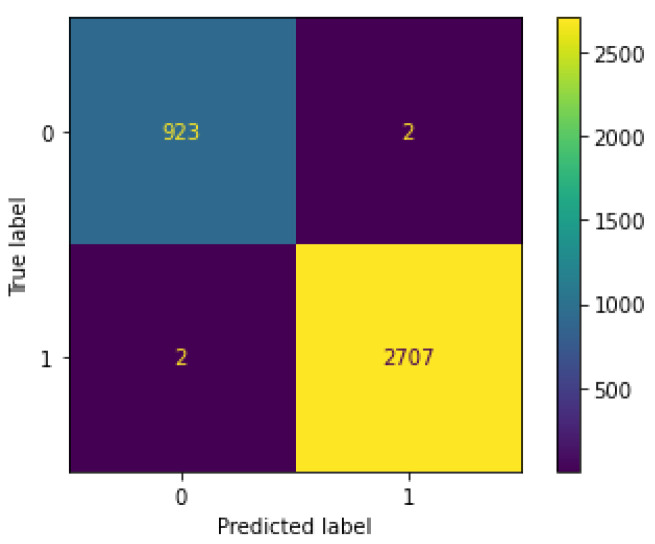
Confusion matrix for Decision Tree binary classification, using the best feature set. The numbers in the axes mean 0 for Normal class, and 1 for Attack class.

From our results, we can see that Decision Tree is the best anomaly detection method for the IDS proposed, as shown in the results for accuracy, precision, recall, F1 score, and time performance, outperforming all the other models in the three feature sets, see Figure 5. We consider the third feature set as the most appropriate one for our novel IDS, since it shows stable results for Machine Learning and Deep Learning models (similar to results in the state of the art), both in multiclass and binary classifications, whilst not using timestamps as learnable features (which can lead to poor performance in a real-time real-world scenario). In addition, results in the literature use the Argus seq as one feature they feed in their models, as our third feature set does. See Figure 6 and Figure 7 for the Decision Tree confusion matrices using this feature set.

Not using neither timestamps nor the Argus sequence number (as in the second feature set), caused the Deep Learning models to have accuracies around 96% and 97% for both binary and multiclass classification, which is lower than the performance achieved by standard ML models. Although initially this result may appear surprising, we argue this is due to the fact that the DL models learn to depend heavily on these particular features. Given the recurrent nature of the neural networks we assessed, these features (as provided in the original dataset) may display strong temporal dependencies (with strong correlations to the categories our models are classifying), once again strengthening the network dependence on these features, leading to poor generalization when implemented with online data. Nonetheless, Random Forest and Decision Tree still show the strongest performance when trained on this feature set, achieving results above 99.8%, which can be explained due to the random nature of these models that allows overcoming dependencies on temporal data. It should also be noted that we did not find other studies that use a similar set of features as that proposed in our second one, so we cannot establish a fair comparison to other works. In addition, the models trained on this feature set are totally independent of temporal characteristics such as timestamps and, particularly, Argus-generated sequence numbers, which make strong generalization models suitable for online IDS implementations.

In addition to all these experiments, binary classification for Normal flows vs. each DDoS/DoS protocol were performed. Table 20, Table 21 and Table 22, show the best anomaly detection models regarding accuracy, precision, recall, and F1 score, for each of those combinations. It can be seen that Decision Tree and Random Forest are the strongest models, achieving 100% across all the metrics in several combinations.

### Comparison with Previous Works

Unlike previous works, this study addresses the class imbalance problem of the Bot-IoT dataset without adding class weights (which can lead to poor generalization, as seen in [14,17]), and without generating synthetic data. With this, we carried out extensive experimentation of normal flows vs. denial of service attacks, in binary and multiclass classifications. Under these analyses, three different feature sets were selected from the original dataset (with larger size and solving the problem of missing information, compared to [13]). We discussed why different flows processors could be used in a real-world scenario and the importance of learning different features.

Likewise, our work shows a comprehensive evaluation of seven distinct Machine Learning and Deep Learning models different to [13,15,16,17], where only either ML or DL models are assessed by the authors. In addition, we applied a systematic tuning of our models hyper parameters and dedicated 10% of our data to validation, in contrast to the process followed in [17]. With respect to performance evaluation, we not only presented confusion matrices and popular metrics (i.e., accuracy, precision, recall, and F1 score), but we also added the time performance measurement to show the IDS feasibility of implementation in production networks, demonstrating that the best resulting models here presented are a realistic solution: this is in contrast to all the related works reviewed in Section 2 that use the Bot-IoT dataset (except for [13]).

Our results match and exceed the current state-of-the-art, with an average accuracy >99% across our three different feature sets, and 100% across several combinations of Normal flows vs. the DDoS/DoS subcategories. These results do not present bias towards a majority class. Compared to the works in our review that deal with class balancing, in [14], the accuracy for multiclass classification for normal flows vs. DDoS and DoS attacks is 99.414%, whilst our best results for the same classification is 99.945% for the first feature set, 99.89% using the second feature set, and 99.917% using the third feature set. In addition, compared to [17], where the stable accuracy was 99% for binary classification in DDoS and DoS attacks protocols and 97% for multiclass classification, we obtain accuracies >99.85% using our 3 different feature sets for binary classification and a best accuracy of at least 99.945% for the multiclass classification on our 3 feature sets.

## 5. Conclusions and Future work

This work uses the Bot-IoT dataset, a state-of-the-art collection of data for protecting IoT networks. The methodology proposed addresses the class imbalance problem of the original dataset (by adding neither synthetic data nor class weights) leading to the creation of a novel IDS based on AI models which focuses on DDoS and DoS attacks. The proposed IDS presents results without biases towards a majority class, achieving an average accuracy >99% with our three distinct feature sets, where the Decision Tree is the outstanding anomaly detection model, whilst being feasible for implementation in real-time production environments, with a remarkable time performance for heavy traffic days (evaluating more than 1681 flows/s). In addition, we achieved 100% across accuracy, precision, recall, and F1 score metrics with the Decision Tree and the Random Forest for several combinations of Normal flows vs. the DDoS/DoS protocols.

As future work, due to the importance of SDN in data centers and in 5G technology and the integration of IoT devices to these networks, we are working on the implementation of both a simulated and a real Software Defined Network infrastructure, using the Open Network Operating System (ONOS) as network controller [4,37], to integrate the IDS developed in this research as a detection service, and we will also integrate a mitigation strategy. The installation and running of this IDS in the SDN controller will solve the issue pointed out in [38], where anomaly detection in heterogeneous sensor networks (as the Bot-IoT testbed) is difficult to achieve directly on the sensor nodes (which have light computing power and limited memory).

Another future direction that is worth mentioning is the interconnection of different IoT environments, considering them as nodes linked by a given relationship and forming a graph, as in a smart city [39]. For this Multiple IoT paradigm (MIoT) [40], it would be interesting to evaluate our proposed IDS because anomalies are less evident in an MIoT than in a single IoT scenario [39].

## Figures and Tables

**Table 1:** Comparison between our approach and the related work around the Bot-IoT dataset.

	This work	[11]	[12]	[13]	[14]	[15]	[16]	[17]
Class balancing	✔	✗	✗	✗	✔	✗	✗	✔
ML models evaluation	✔	✔	✔	✔	✔	✔	✗	✔
DL models evaluation	✔	✔	✔	✗	✔	✗	✔	✗
Feature set(s) proposal	✔	✔	✗	✔	✔	✔	✗	✔
Time performance evaluation	✔	✗	✗	✔	✗	✗	✗	✗
Flow-level detection	✔	✔	✔	✔	✗	✔	✔	✔

**Table 2:** Feature sets selected.

Name	Features	Description
First feature set	stime, pkts, bytes, ltime, seq, dur, mean, stddev, sum, min, max, spkts, dpkts, sbytes, dbytes, rate, srate, drate	Using timestamps, the Argus sequence number, and the statistical variables (i.e., rates, mean, maximum, minimum, etc.).
Second feature set	pkts, bytes, dur, mean, stddev, sum, min, max, spkts, dpkts, sbytes, dbytes, rate, srate, drate	With no timestamps neither the Argus sequence number, only the statistical variables.
Third feature set	pkts, bytes, seq, dur, mean, stddev, sum, min, max, spkts, dpkts, sbytes, dbytes, rate, srate, drate	With the Argus sequence number and the statistical variables.

**Table 3:** Variables description.

Feature	Description
stime	Record start time.
ltime	Record last time.
seq	Argus sequence number.
pkts	Total number of packets in transaction.
bytes	Total number of bytes in transaction.
dur	Record total duration.
mean	Average duration at records aggregate level.
stddev	Standard deviation of the duration at records aggregate level.
sum	Total duration at records aggregate level.
min	Minimum duration at records aggregate level.
max	Maximum duration at records aggregate level.
spkts	Source-to-destination packet count.
dpkts	Destination-to-source packet count.
sbytes	Source-to-destination bytes count.
dbytes	Destination-to-source bytes count.
rate	Total packets per second in transaction.
srate	Source-to-destination packets per second.
drate	Destination-to-source packets per second.

**Table 4:** Summary of ML models parameters for the first feature set.

Model	Binary Classification	Multiclass Classification
SVM	Kernel: Radial Basis Function Max iterations: 70,000	Kernel: Linear Max iterations: 70,000
Decision Tree	Max depth: 11 Entropy criterion	Max depth: 10 Entropy criterion
Random Forest	Max depth: 11 Entropy criterion Trees: 12	Max depth: 10 Entropy criterion Trees: 9

**Table 5:** Summary of ML models parameters for the second feature set.

Model	Binary Classification	Multiclass Classification
SVM	Kernel: Radial Basis Function Max iterations: 50,000	Kernel: Radial Basis Function Max iterations: 50,000
Decision Tree	Max depth: 7 Entropy criterion	Max depth: 8 Entropy criterion
Random Forest	Max depth: 7 Entropy criterion Trees: 2	Max depth: 8 Entropy criterion Trees: 9

**Table 6:** Summary of ML models parameters for the third feature set.

Model	Binary Classification	Multiclass Classification
SVM	Kernel: Radial Basis Function Max iterations: 50,000	Kernel: Radial Basis Function Max iterations: 70,000
Decision Tree	Max depth: 8 Entropy criterion	Max depth: 7 Entropy criterion
Random Forest	Max depth: 8 Entropy criterion Trees: 11	Max depth: 7 Entropy criterion Trees: 21

**Table 7:** Summary of DL models parameters for the three feature sets.

Model	Binary Classification	Multiclass Classification
RNN, LSTM, GRU, MLP	Classes: 2 Batch size: 128 Input size: 18, 15, and 16 Hidden size: 128 (512 for MLP) Layers: 3 (4 for MLP) Sequence length: 1 (None for MLP) Epochs: 100 Optimizer: Adam Loss function: Cross Entropy Learning rate: 0.0011 Device: CPU	Classes: 4 Batch size: 128 Input size: 18, 15, and 16 Hidden size: 128 (512 for MLP) Layers: 3 (4 for MLP) Sequence length: 1 (None for MLP) Epochs: 100 Optimizer: Adam Loss function: Cross Entropy Learning rate: 0.0011 Device: CPU

**Table 8:** Multiclass classification results for the first feature set.

Model	Accuracy	Precision	Recall	F1 Score
Random Forest	99.945%	99.945%	99.945%	99.945%
Decision Tree	99.917%	99.918%	99.917%	99.917%
LSTM	99.862%	99.862%	99.864%	99.863%
GRU	99.862%	99.861%	99.865%	99.863%
MLP	99.862%	99.861%	99.865%	99.863%
RNN	99.807%	99.806%	99.811%	99.808%
SVM	94.056%	94.661%	94.056%	94.122%

**Table 9:** Binary classification results for the first feature set.

Model	Accuracy	Precision	Recall	F1 Score
Random Forest	99.972%	99.973%	99.972%	99.972%
Decision Tree	99.945%	99.945%	99.945%	99.945%
RNN	99.862%	99.889%	99.926%	99.908%
MLP	99.862%	99.889%	99.926%	99.908%
GRU	99.835%	99.852%	99.926%	99.889%
LSTM	99.807%	99.816%	99.926%	99.871%
SVM	98.404%	98.431%	98.404%	98.388%

**Table 10:** Multiclass classification results for the second feature set.

Model	Accuracy	Precision	Recall	F1 Score
Random Forest	99.89%	99.89%	99.89%	99.89%
Decision Tree	99.862%	99.863%	99.862%	99.862%
MLP	96.34%	96.372%	96.375%	96.354%
GRU	96.23%	96.276%	96.25%	96.249%
LSTM	96.01%	96.042%	96.042%	99.022%
RNN	95.019%	95.126%	95.027%	95.049%
SVM	75.482%	78.481%	75.482%	75.218%

**Table 11:** Binary classification results for the second feature set.

Model	Accuracy	Precision	Recall	F1 Score
Decision Tree	99.862%	99.862%	99.862%	99.862%
Random Forest	99.835%	99.835%	99.835%	99.835%
GRU	97.111%	97.797%	98.339%	98.067%
LSTM	96.753%	97.437%	98.228%	97.831%
MLP	96.505%	97.498%	97.822%	97.66%
RNN	96.147%	97.381%	97.453%	97.417%
SVM	81.205%	84.721%	81.205%	76.825%

**Table 12:** Multiclass classification results for the third feature set.

Model	Accuracy	Precision	Recall	F1 Score
Random Forest	99.917%	99.918%	99.917%	99.917%
Decision Tree	99.862%	99.863%	99.862%	99.862%
GRU	99.697%	99.693%	99.702%	99.697%
MLP	99.642%	99.638%	99.646%	99.641%
LSTM	99.56%	99.557%	99.565%	99.561%
RNN	99.56%	99.556%	99.566%	99.56%
SVM	89.351%	89.603%	89.351%	89.347%

**Table 13:** Binary classification results for the third feature set.

Model	Accuracy	Precision	Recall	F1 Score
Decision Tree	99.89%	99.89%	99.89%	99.89%
Random Forest	99.835%	99.835%	99.835%	99.835%
MLP	99.697%	99.742%	99.852%	99.797%
GRU	99.642%	99.595%	99.926%	99.76%
RNN	99.615%	99.595%	99.889%	99.742%
LSTM	99.615%	99.559%	99.926%	99.742%
SVM	94.194%	94.347%	94.194%	94.246%

**Table 14:** Multiclass classification time performance for the first feature set.

Model	Avg Flows/s	Stddev Flows/s
Decision Tree	29,453	790.687
MLP	8306	537.827
SVM	4283	139.935
RNN	4158	59.906
GRU	2497	51.75
LSTM	2388	20.823
Random Forest	1813	65.692

**Table 15:** Binary classification time performance for the first feature set.

Model	Avg Flows/s	Stddev Flows/s
Decision Tree	29,452	716.966
MLP	9411	38.543
SVM	4956	25.011
RNN	4375	77.826
GRU	2661	8.712
LSTM	2610	5.094
Random Forest	1350	81.339

**Table 16:** Multiclass classification time performance for the second feature set.

Model	Avg Flows/s	Stddev Flows/s
Decision Tree	30,362	681.989
MLP	9319	48.97
RNN	4742	49.485
GRU	2864	17.051
LSTM	2702	33.465
Random Forest	1954	15.106
SVM	651	8.033

**Table 17:** Binary classification time performance for the second feature set.

Model	Avg Flows/s	Stddev Flows/s
Decision Tree	29,940	523.611
MLP	9177	142.993
RNN	4697	27.281
Random Forest	4571	60.758
GRU	2763	49.491
LSTM	2687	22.446
SVM	866	7.232

**Table 18:** Multiclass classification time performance for the third feature set.

Model	Avg Flows/s	Stddev Flows/s
Decision Tree	33,094	378.595
MLP	9934	257.982
RNN	4823	99.721
GRU	2918	51.754
LSTM	2877	65.451
SVM	1171	17.393
Random Forest	994	22.931

**Table 19:** Binary classification time performance for the third feature set.

Model	Avg Flows/s	Stddev Flows/s
Decision Tree	32,607	151.361
MLP	10,017	101.06
RNN	4883	123.54
GRU	2996	34.989
LSTM	2864	79.405
Random Forest	1668	89.448
SVM	1422	10.979

**Table 20:** Binary classification results for Normal flows vs. DDoS/DoS subcategories (protocols), using the first feature set.

Classes	Best Model (s)	Accuracy	Precision	Recall	F1 Score
Normal vs. DDoS	Random Forest	99.956%	99.956%	99.956%	99.956%
Normal vs. DDoS UDP	Decision Tree and Random Forest	99.853%	99.853%	99.853%	99.853%
Normal vs. DDoS HTTP	Decision Tree and Random Forest	100%	100%	100%	100%
Normal vs. DDoS TCP	Decision Tree and Random Forest	100%	100%	100%	100%
Normal vs. DoS	Random Forest	99.956%	99.956%	99.956%	99.956%
Normal vs. DoS UDP	All models, except for SVM	100%	100%	100%	100%
Normal vs. DoS HTTP	Decision Tree	100%	100%	100%	100%
Normal vs. DoS TCP	All models, except for SVM	100%	100%	100%	100%

**Table 21:** Binary classification results for Normal flows vs. DDoS/DoS subcategories (protocols), using the second feature set.

Classes	Best Model (s)	Accuracy	Precision	Recall	F1 Score
Normal vs. DDoS	Decision Tree and Random Forest	99.956%	99.956%	99.956%	99.956%
Normal vs. DDoS UDP	Decision Tree and Random Forest	99.853%	99.853%	99.853%	99.853%
Normal vs. DDoS HTTP	Decision Tree and Random Forest	100%	100%	100%	100%
Normal vs. DDoS TCP	Decision Tree and Random Forest	100%	100%	100%	100%
Normal vs. DoS	Random Forest	99.868%	99.868%	99.868%	99.868%
Normal vs. DoS UDP	All models, except for SVM	100%	100%	100%	100%
Normal vs. DoS HTTP	Decision Tree	100%	100%	100%	100%
Normal vs. DoS TCP	Decision Tree and Random Forest	100%	100%	100%	100%

**Table 22:** Binary classification results for Normal flows vs. DDoS/DoS subcategories (protocols), using the third feature set.

Classes	Best Model (s)	Accuracy	Precision	Recall	F1 Score
Normal vs. DDoS	Random Forest	99.956%	99.956%	99.956%	99.956%
Normal vs. DDoS UDP	Random Forest	99.853%	99.853%	99.853%	99.853%
Normal vs. DDoS HTTP	Decision Tree and Random Forest	100%	100%	100%	100%
Normal vs. DDoS TCP	Random Forest	100%	100%	100%	100%
Normal vs. DoS	Random Forest	99.868%	99.868%	99.868%	99.868%
Normal vs. DoS UDP	All models, except for SVM	100%	100%	100%	100%
Normal vs. DoS HTTP	Decision Tree	100%	100%	100%	100%
Normal vs. DoS TCP	All models, except for SVM	100%	100%	100%	100%

## Data Availability

A publicly available dataset was analyzed in this study. This data can be found here: https://research.unsw.edu.au/projects/bot-iot-dataset (accessed on 26 January 2021).
